# Cardiac Compression of Lung Lower Lobes after Coronary Artery Bypass Graft with Cardiopulmonary Bypass

**DOI:** 10.1371/journal.pone.0078643

**Published:** 2013-11-11

**Authors:** Flávio H. Neves, Maria J. Carmona, José O. C. Auler, Roseny R. Rodrigues, Jean Jacques Rouby, Luiz M. S. Malbouisson

**Affiliations:** 1 Divisão de Anestesia, Hospital das Clínicas da Faculdade de Medicina da Universidade de São Paulo, São Paulo, Brazil; 2 Multidisciplinary Intensive Care Unit, Department of Anesthesiology, Hôpital de la Pitié-Salpêtrière, University Pierre et Marie Curie, Paris, France; University of Otago, New Zealand

## Abstract

**Background:**

Atelectasis is a major cause of hypoxemia after coronary artery bypass grafting (CABG) and is commonly ascribed to general anesthesia, high inspiratory oxygen concentration and cardiopulmonary bypass (CPB). The objective of this study was to evaluate the role of heart-induced pulmonary compression after CABG with CPB.

**Methods:**

Seventeen patients without pre-operative cardiac failure who were scheduled for coronary artery bypass graft underwent pre- and postoperative thoracic computed tomography. The cardiac mass, the pressure exerted on the lungs by the right and left heart and the fraction of collapsed lower lobe segments below and outside of the heart limits were evaluated on a computed tomography section 1 cm above the diaphragmatic cupola.

**Results:**

In the postoperative period, cardiac mass increased by 32% (117±31 g versus 155±35 g, p<0.001), leading to an increase in the pressure that was exerted on the lungs by the right (2.2±0.6 g.cm^−2^ versus 3.2±1.2 g.cm^−2^, p<0.05) and left heart (2.4±0.7 g.cm^−2^ versus 4.2±1.8 g.cm^−2^, p<0.001). The proportion of collapsed lung segments beneath the heart markedly increased [from 6.7% to 32.9% on the right side (p<0.001) and from 6.2% to 29% on the left side (p<0.001)], whereas the proportion of collapsed lung segments outside of the heart limits slightly increased [from 0.7% to 10.8% on the right side (p<0.001) and from 1.5% to 12.6% on the left side (p<0.001)].

**Conclusion:**

The pressure that is exerted by the heart on the lungs increased postoperatively and contributed to the collapse of subjacent pulmonary segments.

## Introduction

Atelectasis is a highly prevalent pulmonary complication in patients undergoing cardiac surgery with cardiopulmonary bypass (CPB) and an important cause of postoperative hypoxemia. [1]–[3] Pulmonary collapse occurs early after the induction of anesthesia and persists for several days postoperatively. [4]–[7] Studies based on thoracic computed tomography (CT) have shown that pulmonary collapse is mainly distributed to the dependent regions close to the diaphragm and may encompass up to 35% of the overall lung parenchyma. [2], [8]–[10] Some mechanisms have been proposed to explain the pulmonary collapse in patients undergoing cardiac surgery, such as the use of high inspiratory oxygen fraction, [11] cephalic displacement of the relaxed diaphragm compressing the caudal portions of the lower lobes, [12], [13] surgical manipulation of pulmonary structures and depressurization of the respiratory system during CPB to enable better visualization of the surgical field. However, despite the fact that most of the mechanisms leading to intraoperative pulmonary collapse vanish when patients awake and resume spontaneous breathing, postoperative atelectasis and hypoxemia may persist for several days [5], [14].

Little is known about the contribution of heart-induced pulmonary compression to the development and perpetuation of dependent pulmonary atelectasis following cardiac surgery with CPB. In the supine position, large segments of the lower lobes are located beneath the heart [15], and previous reports have described the mechanical compression of the airways by an enlarged left atrium in neonates, infants and adult patients, leading to extended atelectasis. [16]–[18] Furthermore, radioisotopic ventilation–perfusion studies have demonstrated reduced lower lobe ventilation in patients with cardiomegaly [19], [20], a phenomenon that is reversible with prone positioning. [21] This result is highly suggestive of airway closure or narrowing or alveolar collapse. Using quantitative thoracic CT analysis, Malbouisson et al. demonstrated that the pressure exerted by the cardiac mass on the lower lobes was implicated in the regional loss of pulmonary aeration in patients with acute respiratory distress syndrome (ARDS) [15].

In patients undergoing coronary artery bypass, systemic inflammatory response, positive fluid balance and cardiac wall edema may contribute to an increase in the weight of the heart, thereby augmenting heart-induced pulmonary compression. The objective of this study was to evaluate the contribution of the cardiac mass to postoperative atelectasis in patients undergoing coronary artery bypass grafting (CABG) with CPB.

## Methods

This study was approved by an institutional ethics committee (Comissão de Ética para Análise de Projetos de Pesquisa do Hospital das Clínicas da Faculdade de Medicina da Universidade de São Paulo – CAPPESq - number 756/06), and informed consent was obtained from all participants after the study protocol was fully explained. Twenty patients who were scheduled for surgical treatment of coronary artery disease were included in this prospective study. All patients had normal preoperative left ventricular function, characterized by ejection fractions greater than 50%, and were submitted to pre- and postoperative whole-lung CT scans. Exclusion criteria were as follows: chronic obstructive pulmonary disease, preoperative pulmonary congestion, the need for any form of positive pressure ventilator support in the first postoperative day, transfusion of more than 3 packed red cell units and perioperative hemodynamic instability requiring vasopressors.

### Perioperative Patient Care, Hemodynamics and Blood Gas Measurements

General anesthesia was induced with fentanyl, etomidate and atracurium and was maintained with isoflurane, fentanyl and pancuronium bromide. During the surgical procedure, patients were monitored with electrocardiography, capnography, pulse oximetry, esophageal temperature and an invasive arterial pressure line. After anesthesia induction, a fiberoptic thermodilution pulmonary artery catheter (CCO/SvO2/VIPTMTD catheter - Edwards Lifesciences, Irvine, CA, USA) was placed through the right internal jugular vein. Mechanical ventilator settings throughout the study were as follows: 1) tidal volume of 8 mL.kg^−1^; 2) respiratory rate to maintain PaCO_2_ values between 35 and 40 mmHg; 3) inspiratory time of 33% of the total respiratory cycle time; 4) inspired oxygen fraction of 0.6; and 5) positive end-expiratory pressure (PEEP) of 5 cmH_2_O.

Before vascular cannulation, 400 U.kg^−1^ of heparin was administered intravenously. Aortic bi-caval CPB was performed using an extracorporeal circuit and a membrane oxygenator (Braile, São José do Rio Preto, SP, Brazil) that was primed with 1500 mL of lactated Ringer solution, mannitol 1 g.kg^−1^ and heparin 10,000 UI. Cardiac protection was achieved using hypokalemic, hypothermic blood cardioplegia solution. During CPB, mechanical ventilation was stopped. Coronary grafts were performed at the discretion of the surgeon. Vasoactive drugs were infused to facilitate CPB weaning according to the attending anesthesiologist’s decision. When mechanical ventilation was resumed at the end of CPB, a lung recruitment maneuver was performed using an airway pressure of 30 cmH_2_O, which was applied for 15 seconds. The aim was to fully re-expand the lungs that had collapsed following interruption of the mechanical ventilation and surgical manipulations.

In the intensive care unit (ICU), patients were allowed to wake up and were extubated as soon as they could spontaneously breathe with a respiratory rate <30.min^−1^ and maintain a PaO_2_>80 mm Hg at FiO_2_ 0.4 and a spontaneous tidal volume >5 mL.kg^−1^. After extubation, oxygen was supplemented to the patients with a Venturi mask that delivered known oxygen concentrations. Postoperative pain was prevented by tramadol combined with morphine boluses whenever necessary. Non-invasive positive pressure ventilation was not used postoperatively. According to institutional standard care protocol, hemodynamic measurements, including analysis of the arterial and venous blood samples, were performed after intubation, after CPB, at ICU admission and 24 h after surgery. In addition, a baseline arterial blood sample was collected before anesthesia induction.

### Thoracic CT Scan Protocol

#### Acquisition of the CT sections

On the day before surgery, a fast spiral thoracic CT scan (Toshiba Aquillion 16 CT scanner, Toshiba Medical Division Japan) was obtained. Thoracic CT scanning was performed from the apex to the diaphragm, and the exposures were taken at 120 kV and 250 mAs. During image acquisition, the patients were asked to hold their breath at the end of a normal expiration for 15 to 20 seconds to acquire the volumetric CT images at functional residual capacity. To reach this specific respiratory condition, the patients were instructed about the respiratory hold maneuver and repeated it at least 10 times before thoracic CT scanning. During the entire procedure, electrocardiography, non-invasive arterial pressure and pulse oximetry were monitored using a Philips M3 transport monitor (Philips, Eindhoven, The Netherlands). A second CT was obtained in the same respiratory condition during the first postoperative day, approximately 24 h following admission in the ICU and extubation. All volumetric tomographic studies were reconstructed by the CT scan console as 10-mm-thick contiguous *Digital Imaging and Communications in Medicine* (DICOM) images. Images were recorded in the hospital’s Picture Archiving and Communication System and were transferred to a personal computer for quantitative CT image analysis.

### Measurement of Thoracic and Cardiac Dimensions

**Figure 1:**
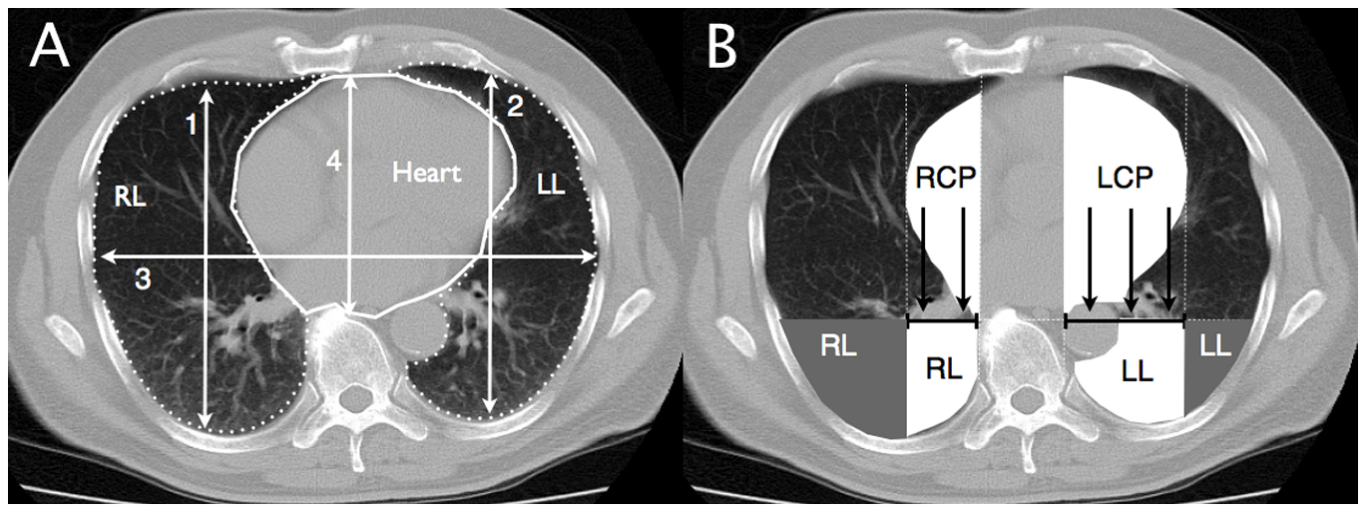
Figure shows a schematic representation of the CT image parameters analyzed. Panel A shows rib cage dimensions (numbered white arrows), cardiac silhouette (continuous white line contour) and lung boundaries (dotted white line contours) on a representative CT section located 1 cm above the diaphragm. Numbers 1 and 2 identify right and left anteroposterior sagittal distances, number 3 the maximal thoracic transverse distance, number 4 the sternovertebral distance and number 5 the cardiac transverse diameter (dashed line). Panel B shows a schematic representation of the orthogonal pressure (black arrows) exerted by the left (LCP) and right (RCP) cardiac protrusions (white cardiac segments) on subjacent surface (tipped black solid lines) of lower lobes located beneath the heart (white lung areas). Parts of lower lobes located outside of heart limits are represented in gray.

The sternovertebral distance, the maximal thoracic transverse distance and the left and right anteroposterior sagittal distances were measured using the caliper tool of a software program designed to analyze radiological DICOM images (Osiris 4.19, HUG, Geneva, Switzerland) in each individual 10-mm-thick CT section (figure 1A). Left and right cephalocaudal thoracic dimensions were measured by counting the number of 10-mm-thick CT sections that were reconstructed from the pulmonary apex to the diaphragm. In the CT sections located 1, 2 and 3 cm above the diaphragm, the maximal horizontal diameters of the cardiac silhouette and of the right and left cardiac protrusions, defined as the segments of the cardiac silhouette located above the left and right lower lobes, were measured, as shown in figure 1B.

### Assessment of Regional Pulmonary Volume and Lung Tissue Mass Present in the CT Section Located 1 cm above the Diaphragm

On the CT section located 1 cm above the diaphragm, the volumes of gas and tissue of the left and right lungs were analyzed as described previously. ^15,22^ Briefly, after manually delineating the external boundaries of the left and right lung parenchyma using the Osiris software (figure 1A), pixels contained within each pulmonary region of interest were distributed on 1200 compartments according to their X-ray attenuation coefficient (CT number). For each pixel, the CT number represents the attenuation coefficient of the X-ray by the structure being studied minus the attenuation coefficient of water, divided by the attenuation coefficient of water expressed in Hounsfield units (HU). For each compartment of a known CT number, the volume and mass were computed according to the following formulas:
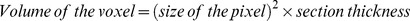
(1)

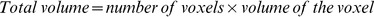
(2)

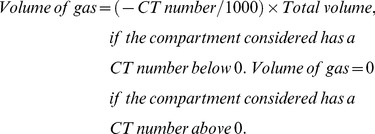
(3)

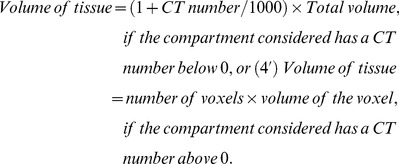
(4)

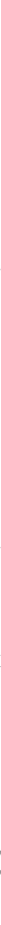
(5)


Lung tissue mass and pulmonary volumes of each CT section or region of interest were computed by adding all of the partial masses and volumes of the compartments that were present within the CT section. The calculation of the mass is based on the tight correlation existing between radiological and physical densities. [15], [22] The distribution of lung aeration within each CT section or region of interest used the following classification: 1) hyperinflated regions characterized by CT numbers between −1000 HU and −900 HU [23], 2) normally aerated regions characterized by CT numbers between −900 HU and −500 HU [24], 3) poorly aerated regions characterized by CT numbers between −500 HU and −100 HU and 4) non aerated regions characterized by CT numbers between −100 and +100 HU) [25], [26].

### Determination of Volume and Mass of the Heart and the Pressure Exerted by the Heart over the Subjacent Lung Segments

The volume and mass of the heart were determined on the CT section located 1 cm above the diaphragm using the same method as described in the previous section. In the first step, the entire cardiac silhouette was manually delineated, as shown in figure 1. The pixels within this area were distributed along 256 compartments located between −100 HU and 200 HU, each compartment representing a 1-HU interval. The volume and mass of the heart were computed by adding partial masses and volumes of the measured compartments, according to formulas 4 and 5.

In a second step, the volume and the mass of the left and right cardiac protrusions located above the lungs in the supine position were determined (figure 1). The mean pressure that was exerted by the heart on the subjacent segments of the lower lobes was obtained by dividing the mass of the cardiac areas located above the left and right lower lobes by the linear surface corresponding to the maximal width of each cardiac protrusion. The delineations performed in the preoperative CT images were transposed to the postoperative images, and anatomical landmarks defining the delineation, such as vessels and bronchi, were checked to ensure that the same lung segments were enclosed in the given region of interest.

### Evaluation of the Impact of Pressure Exerted by the Cardiac Segments on the Subjacent Pulmonary Segments

To evaluate the influence of the pressure exerted by each cardiac protrusion on the subjacent pulmonary segments, the lowermost parts of the left and right lungs were divided into two segments, as shown in the left panel of figure 1: 1) the pulmonary segment below the heart, shown as the white areas below the cardiac protrusions, and 2) the pulmonary segment outside of the heart limits, shown as the gray areas (figure 1). The role played by heart-induced mechanical compression was assessed by comparing the aeration of pulmonary regions within and outside the heart limits. In each region, the volume of gas and tissue and the distribution of aeration were computed using the same approach as described in the section “Analysis of the pulmonary volumes and mass on the CT image located 1 cm above the diaphragm”.

To account for the increase in lung weight secondary to high-permeability type postoperative pulmonary edema [2], [27], the aeration of pulmonary segments located below the heart and outside of the heart limits in the pre- and postoperative thoracic CT scans was expressed as the fraction of non-aerated to overall region lung mass.

### Statistical Analysis

The sample size was calculated to detect a 30% postoperative increase in the mean pressure exerted by the heart on the lower lobes compared to the preoperative values, assuming a standard deviation of 30% of the mean, with a p-value less than 0.05 and a power of 0.90 (Heinrich-Heine-Universitat Dusseldorf, Dusseldorf, Germany). All variables were tested for normal distribution using Kolmogorov-Smirnov, D’Agostino and Pearson omnibus normality and Shapiro-Wilk tests. Hemodynamic and metabolic measurements were examined over time using one-way analysis of variance for repeated measures. The overall cardiac mass, different pulmonary volumes and masses of the left and right lungs and the left and right lower lobe segments located beneath and outside of the heart protrusion limits were compared in the preoperative and postoperative CT images using a paired Student T test or the Wilcoxon matched-pairs signed-rank test when indicated. The cardiac and thoracic dimensions, the mass and the pressure exerted by the left and right cardiac protrusions on the subjacent pulmonary parenchyma were compared between pre- and postoperative moments using a two-way analysis of variance for repeated measures. Because the collapsed pulmonary parenchyma fraction was not normally distributed, the comparison between the lower lobe segments located beneath and outside of the heart protrusion limits during pre- and postoperative moments was performed using the Wilcoxon matched-pairs signed-rank test. The post-hoc analysis following the analysis of variances was performed using the Bonferroni method. The statistical analysis was performed using the SPSS 20 statistical package (SPSS Inc., Chicago, IL, USA). All of the parametric data were expressed as means ± SD, unless otherwise specified. Boxplot whiskers were plotted according to the Tukey method. [28] The statistical significance level was fixed at 0.05.

## Results

### Clinical Characteristics of Patients

From the 20 patients who were initially enrolled in the study, 3 were excluded due to the presence of respiratory motion artifacts on CT scans that rendered an accurate analysis impossible. Individual characteristics of the 17 patients who were enrolled in the study are presented in table 1. During the intraoperative period, the patients received 4.227±1.039 mL of crystalloids and 909±202 mL of 6% hydroxyethil starch solution. From the total fluid infused intraoperatively, 2.433±1.806 mL was given during CPB. Dobutamine (8±3 µg.kg^−1^. min) was infused in 15 patients to facilitate CPB weaning and was stopped within 12 hours. In the first postoperative 24 hours, a mean volume of 1550±257 mL crystalloid solution was administered.

**Table 1:** Individual characteristics of the patients undergoing Coronary Artery Bypass Graft with Cardiopulmonary Bypass.

Subjects	Age (y)	Gender	Height (cm)	Weight (kg)	LVEF (%)	CPB duration (min)	Surgery duration (min)
1	48	M	162	66	62	110	450
2	50	M	175	78	55	96	390
3	51	F	185	112	50	100	360
4	62	M	170	70	73	125	370
5	77	M	171	90	70	100	330
6	65	M	170	72	57	142	350
7	57	M	170	81	50	82	315
8	49	M	154	74	55	80	350
9	74	F	180	89	50	75	330
10	73	M	165	75	78	45	375
11	61	F	164	62	50	110	360
12	72	M	165	83	55	90	415
13	61	M	172	72	60	50	325
14	71	M	172	74	69	95	318
15	52	M	158	63	58	93	420
16	70	M	153	70	57	78	360
17	56	F	167	70	70	100	360

Definition of abbreviations: LVEF = left ventricular ejection fraction; M = male; F = female; CPB = cardiopulmonary bypass.

As shown in table 2, there were significant increases in the cardiac index and heart rate during the observation period, while the mean arterial pressure (MAP) transiently decreased after CPB. Central venous and pulmonary capillary wedge pressures and the pulmonary vascular resistance index remained within the normal limits. Twenty-four hours following surgery, the systemic vascular resistance index was significantly lower than the baseline measurement. Serum lactate concentrations were slightly significantly elevated after the end of CPB, but they returned to baseline levels during the first postoperative day. Induction of general anesthesia induced a reduction in the PaO_2_/FiO_2_ ratio that reached its nadir after CPB and returned to post-anesthesia conditions by the 24^th^ postoperative hour, but it remained significantly lower than preoperative values (table 2). All of the patients were extubated within 8 hours from ICU admission. None of the patients developed respiratory failure after extubation, and all were discharged from the ICU to the ward within 72 hours and from the hospital within 10 days after surgery.

**Table 2:** Hemodynamic and metabolic profiles during surgery and in the first 24 postoperative hours.

	Preoperative	After anesthesia	After CPB	ICU admission	ICU 24 h	p value
HR (bpm)	–	69±20	98±17*	98±20*	98±15*	<0.001
MAP (mmHg)	–	74±9	66±8*	84±15	81±9	<0.001
MPAP (mmHg)	–	25±6	27±7	25±7	23±6	0.16
CVP (mmHg)	–	14±6	16±6	14±5	12±4	0.17
PAOP (mmHg)	–	16±4	17±5	15±5	15±4	0.17
CI (L.min^−1^.m^2^)	–	2.5±0.8	3.9±1.4*	3.2±1.1*	3.9±0.7*	0.003
SVRi (dyn.s.cm^−5^.m^2^)	–	2090±667	1255±627*	1944±676	1499±323*	<0.001
PVRi (dyn.s.cm^−5^.m^2^)	–	312±220	225±158	239±129	168±93	0.09
PaO_2_/FiO_2_	403±36	307±85#	230±88* #	265±66* #	295±44#	<0.001
SvO2 (%)	–	80±6	78±10	74±11	69±7	0.09
Lactate (mg.mL^−1^)	12.4±3.4	14.2±3.2	37.4±20.3* #	36.2±27* #	16±6.5	<0.001

Definition of abbreviations: CI = cardiac index; CPB = cardiopulmonary bypass; CVP = central venous pressure; HR = heart rate; MAP = mean arterial pressure; MPAP = mean pulmonary artery pressure; PAOP = pulmonary artery occlusion pressure; PVRi = pulmonary vascular resistance index; SVRi = systemic vascular resistance index. Normal values for lactate <18 mg.dL^−1^. Data are expressed as mean ± standard deviation. The variables were compared by means of one-way ANOVA for repeated measures.

* means p<0.05 compared to after anesthesia.

#means p<0.05 compared to preoperative period.

### Effects of Surgery on Thoracic and Cardiac Dimensions

**Figure 2:**
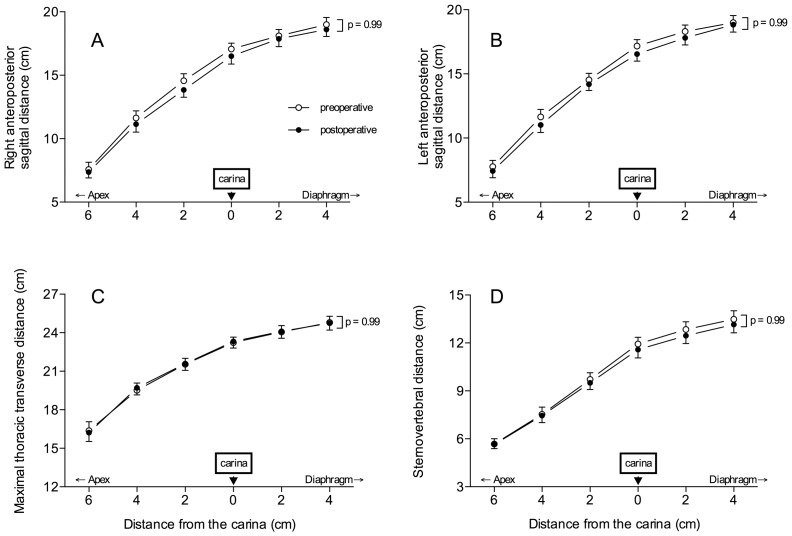
Right and left sagittal distances (panels A and B), maximal thoracic transverse distance (panel C) and sternovertebral distance (panel D) measured on 6 CT sections before (open circles) and after surgery (closed circles). Data are expressed as mean ± SEM.

**Figure 3:**
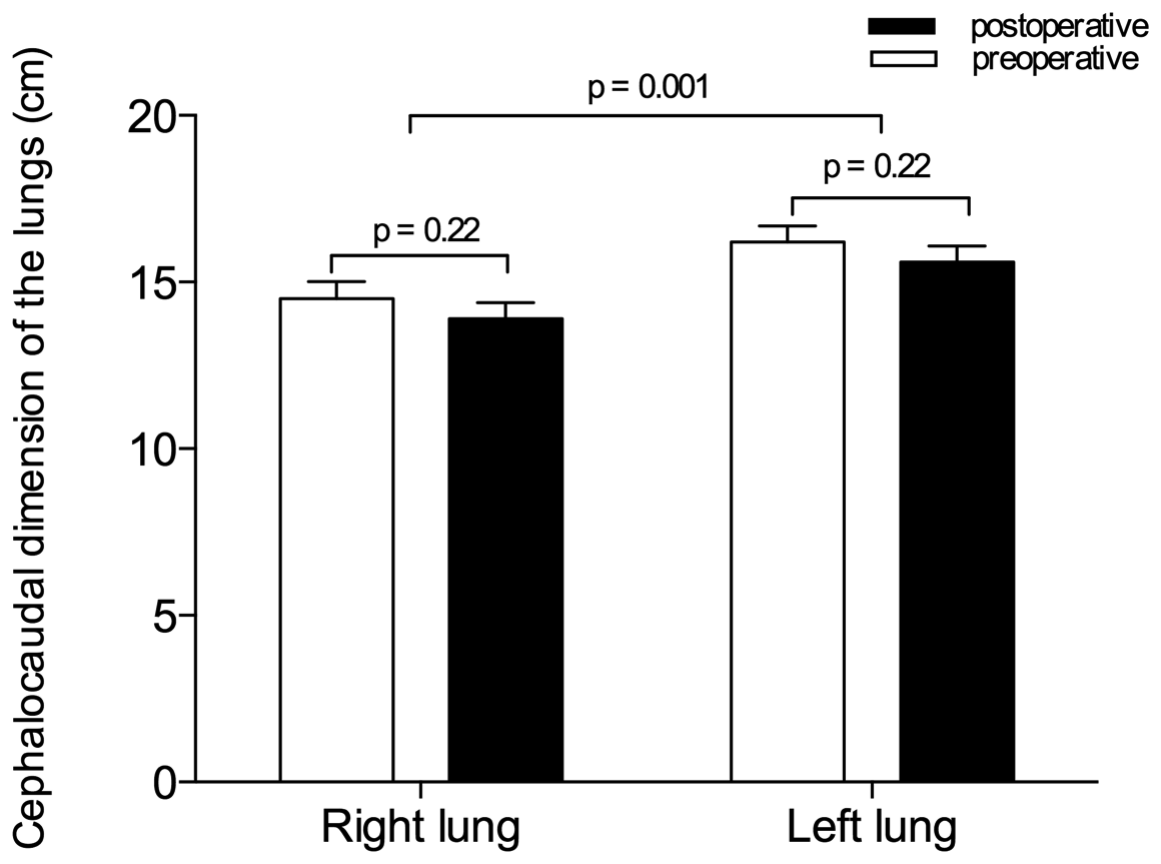
Cephalocaudal dimensions of the lungs (distance from pulmonary apex to the diaphragmatic cupola) measured on 6 CT sections before (open bars) and after surgery (black bars). Data are expressed as mean ± SEM.

As shown in figure 2, sternovertebral, maximal transverse and anteroposterior sagittal dimensions of the rib cage did not change between pre- and postoperative periods. As previously demonstrated, [29], [30] the cephalocaudal distance between the apex and the diaphragm was greater in the left hemi-thorax than in the right hemi-thorax (p = 0.001). However, these dimensions did not vary between pre- and postoperative periods (figure 3).

**Figure 4:**
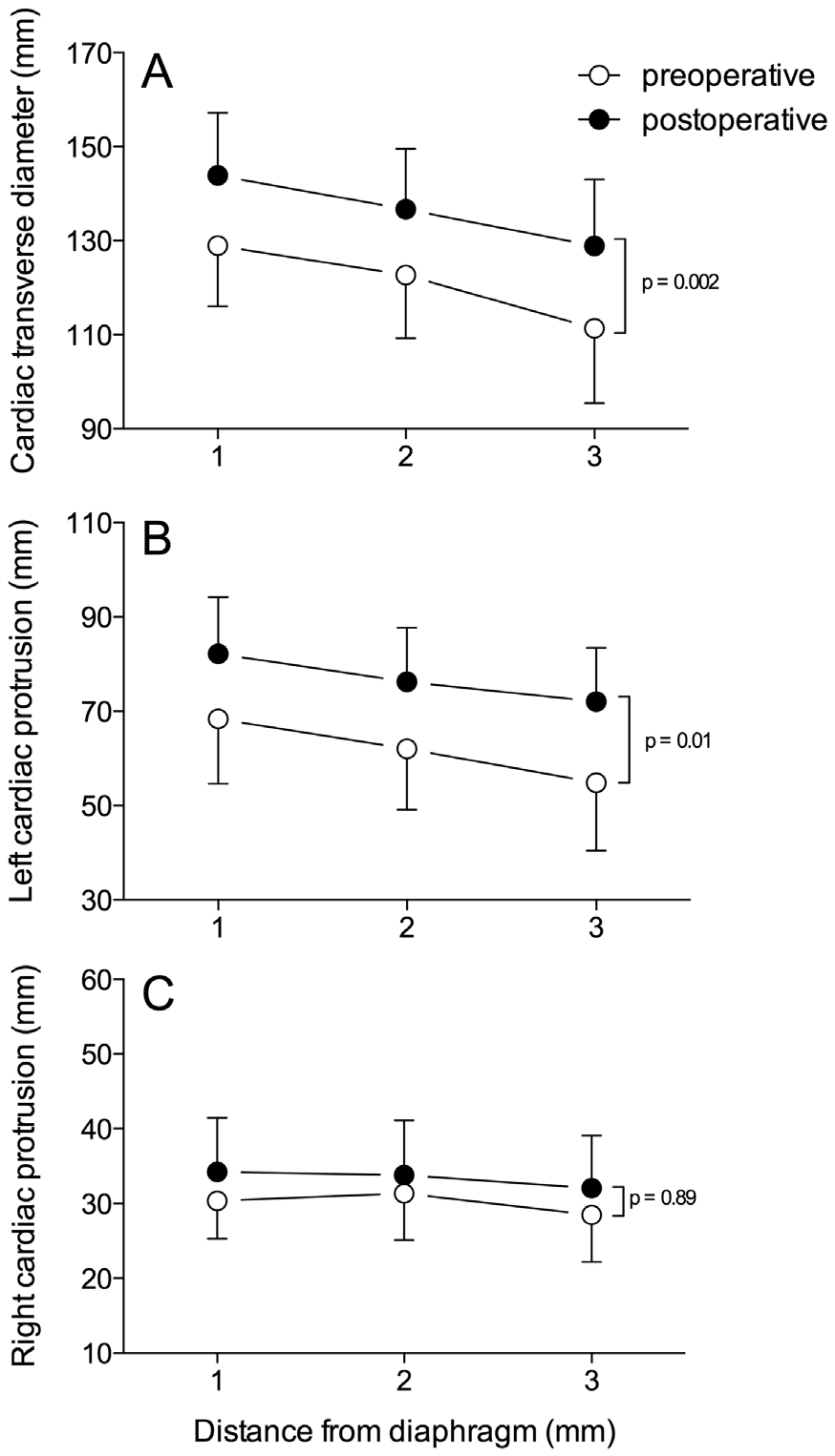
Maximal transverse cardiac distance (panel A), left cardiac protrusion (panel B) and right cardiac protrusion (panel C) measured on 3 CT sections before (open circles) and after surgery (closed circles). Data are expressed as mean ± SEM.

**Figure 5:**
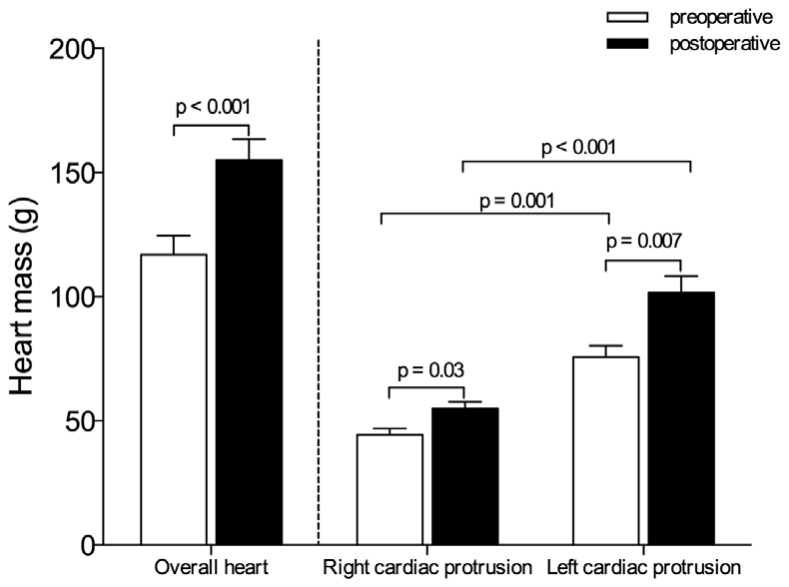
Overall heart, right cardiac protrusion and left cardiac protrusion masses computed before (open bars) and after surgery (black bars). Data are expressed as mean ± SEM.

As illustrated in figure 4, cardiac dimensions significantly increased between pre- and postoperative periods. The transverse cardiac and left cardiac protrusion diameters, measured in 3 contiguous CT sections above the diaphragm, increased when compared to preoperative values (p = 0.002). As shown in figure 5, the postoperative overall heart and the right and left cardiac protrusion masses increased by 32%, 22% and 34%, respectively, when compared to preoperative values (p<0.05). The cardiac protrusion mass was significantly greater on the left than on the right side before and after surgery (p<0.01). Following surgery, the proportion of increase was similar on both sides.

### Changes in Lung Morphology Resulting from Cardiac Surgery

Following CABG with CPB, 32% and 28% decreases in gas volume of right and left lungs (p = 0.001) were measured on the CT section located 1 cm above the diaphragm (table 3). This decrease was associated with 47% and 59% increases in tissue volume (p<0.001). As a result of these opposite changes, no significant variations were observed in the overall volumes of the right and left lungs. Increases in parenchymal mass were measured in the non-aerated and poorly aerated compartments (p<0.001), while the mass of normally aerated parenchyma decreased.

**Table 3:** Overall gas and tissue volumes and mass of right and left lungs measured on the CT section located 1 cm above the diaphragm.

		Before surgery	After surgery	p value
Overall volume (mL)	Right lung	115±34	105±39	0.2
	Left lung	116±35	114±34	0.69
Tissue volume (mL)	Right lung	35±10	51±16	<0.001
	Left lung	34±9	54±15	<0.001
Gas Volume (mL)	Right lung	80±28	54±28	<0.001
	Left lung	83±30	60±30	0.001
Overall parenchyma mass (g)	Right lung	35±10	52±16	<0.001
	Left lung	34±9	54±15	<0.001
Non-aerated parenchyma mass (g)	Right lung	4±6	20±11	<0.001
	Left lung	3±3	23±16	<0.001
Poorly-aerated parenchyma mass (g)	Right lung	8±5	13±4	0.004
	Left lung	9±7	13±6	0.04
Normally-aerated parenchyma mass (g)	Right lung	23±7	18±8	0.03
	Left lung	23±8	18±8	0.04
Hyperinflated parenchyma mass (g)	Right lung	0.1 (0–0.6)	0 (0–0.2)	0.08
	Left lung	0.1 (0–0.7)	0 (0–0.2)	0.35

Data are expressed as mean ± SD or as median (25%–75%). Comparisons were performed by means of paired Student’s t test or Wilcoxon matched-pairs signed rank test when indicated.

### Impact of Heart-induced Mechanical Compression on the Aeration of Lower Lobes

**Figure 6:**
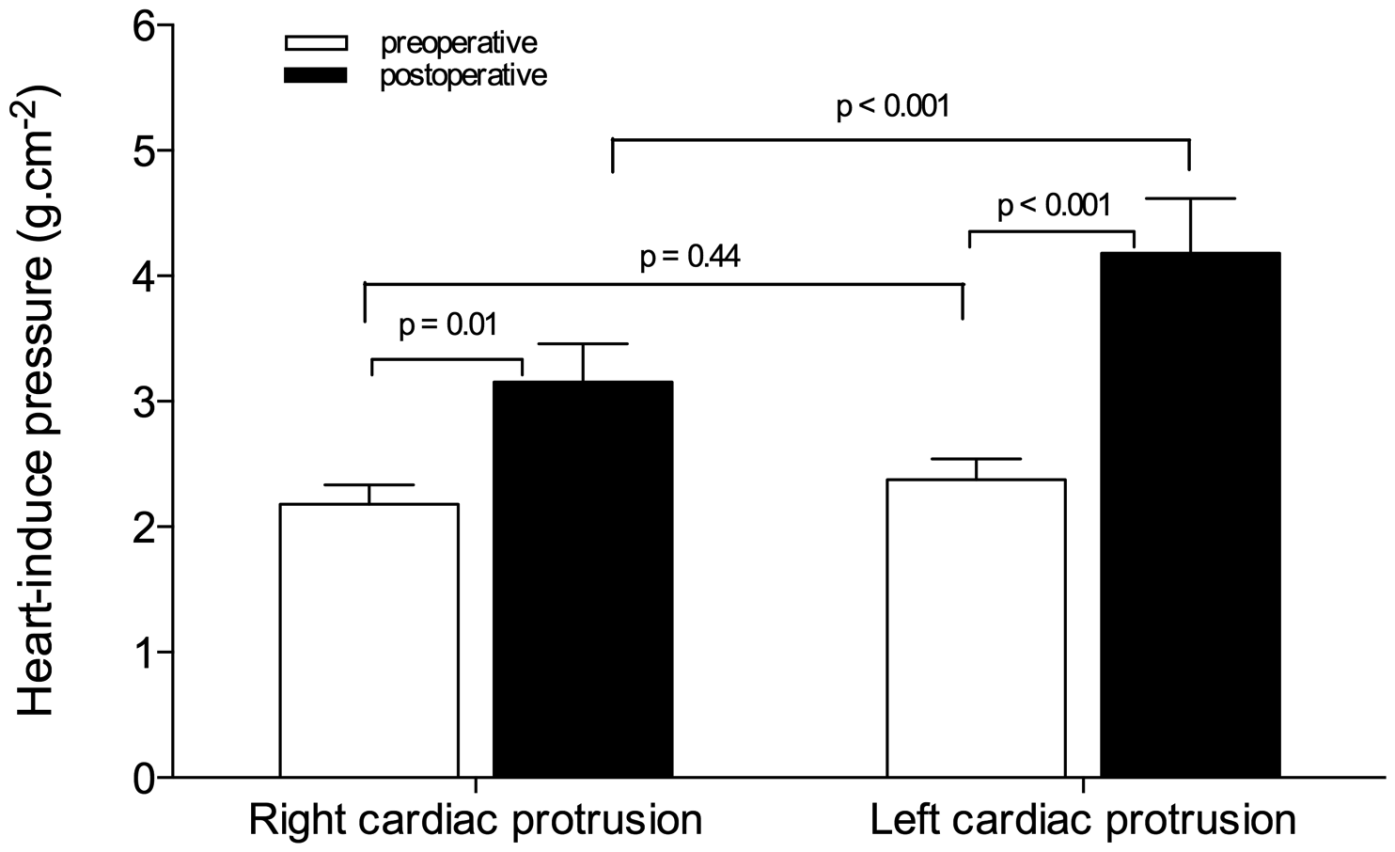
Pressure exerted by the right and left cardiac protrusion masses on subjacent lower lobes measured before (open bars) and after surgery (black bars). Data are expressed as mean ± SEM.

As shown in figure 6, the pressures that were exerted by the right and left heart on subjacent lower lobes before surgery were similar (2.2±0.6 g.cm^2^ and 2.4±0.7 g.cm^2^, respectively). During the first postoperative day, the pressures that were exerted by the right and left cardiac protrusions on the lower lobes increased by 45% and 75%, respectively (p<0.05). The cardiac protrusion-induced pressure was significantly greater in the left than in the right side. Tables 4 and 5 show changes in lung morphology observed in the right and left lower lobe segments located below the cardiac protrusions and outside of the heart limits. Following surgery, the tissue volumes significantly increased in the pulmonary segments below the heart and outside of the heart limits, while the gas volumes significantly decreased in the right and left lower lobes. The normally aerated compartment only decreased significantly in pulmonary segments that were located below the cardiac protrusions; it remained unchanged in the pulmonary segments outside of the heart limits.

**Table 4:** Overall, gas and tissue volumes and mass of right lower lobe segments below and outside of the heart limits measured on the CT section located 1 cm above the diaphragm.

		Before surgery	After surgery	p value
Overall volume (mL)	Below	32±11	35±15	0.39
	Outside	51±27	43±26	0.16
Tissue volume (mL)	Below	13±6	22±7	<0.001
	Outside	14±7	19±12	0.04
Gas Volume (mL)	Below	19±7	38±22	0.01
	Outside	13±11	24±16	0.008
Overall parenchyma mass (g)	Below	13±6	22±7	<0.001
	Outside	14±7	19±12	0.004
Non-aerated parenchyma mass (g)	Below	2±2	12±6	<0.001
	Outside	1±1	6±7	<0.001
Poorly-aerated parenchyma mass (g)	Below	4±3	6±2	0.005
	Outside	2±2	4±2	0.02
Normally-aerated parenchyma mass (g)	Below	7±3	4±4	0.02
	Outside	10±5	9±5	0.24
Hyperinflated parenchyma mass (g)	Below	0 (0–0)	0 (0–0)	0.35
	Outside	0 (0–0.3)	0 (0–0)	0.1

Data are expressed as mean ± SD or as median (25%–75%). Comparisons were performed by means of paired Student’s t test or Wilcoxon matched-pairs signed rank test when indicated.

**Table 5:** Overall gas and tissue volumes and mass of left lower lobe segments below and outside of the heart limits measured on the CT section located 1 cm above the diaphragm.

		Before surgery	After surgery	p value
Overall volume (mL)	Below	35±18	34±16	0.88
	Outside	54±21	49±21	0.22
Tissue volume (mL)	Below	13±5	24±13	0.001
	Outside	16±8	21±9	0.04
Gas Volume (mL)	Below	22±14	10±7	0.007
	Outside	38±16	28±15	0.002
Overall parenchyma mass (g)	Below	13±5	24±13	0.001
	Outside	16±8	21±9	0.04
Non-aerated parenchyma mass (g)	Below	2±2	15±12	<0.001
	Outside	1±2	6±6	0.002
Poorly-aerated parenchyma mass (g)	Below	3±2	6±3	0.03
	Outside	3±3	5±4	0.08
Normally-aerated parenchyma mass (g)	Below	7±4	3±3	0.001
	Outside	12±5	9±5	0.16
Hyperinflated parenchyma mass (g)	Below	0 (0–0)	0 (0–0)	0.39
	Outside	0 (0–0.1)	0 (0–0)	0.12

Data are expressed as mean ± SD or as median (25%–75%). Comparisons were performed by means of paired Student’s t test or Wilcoxon matched-pairs signed rank test when indicated.

**Figure 7:**
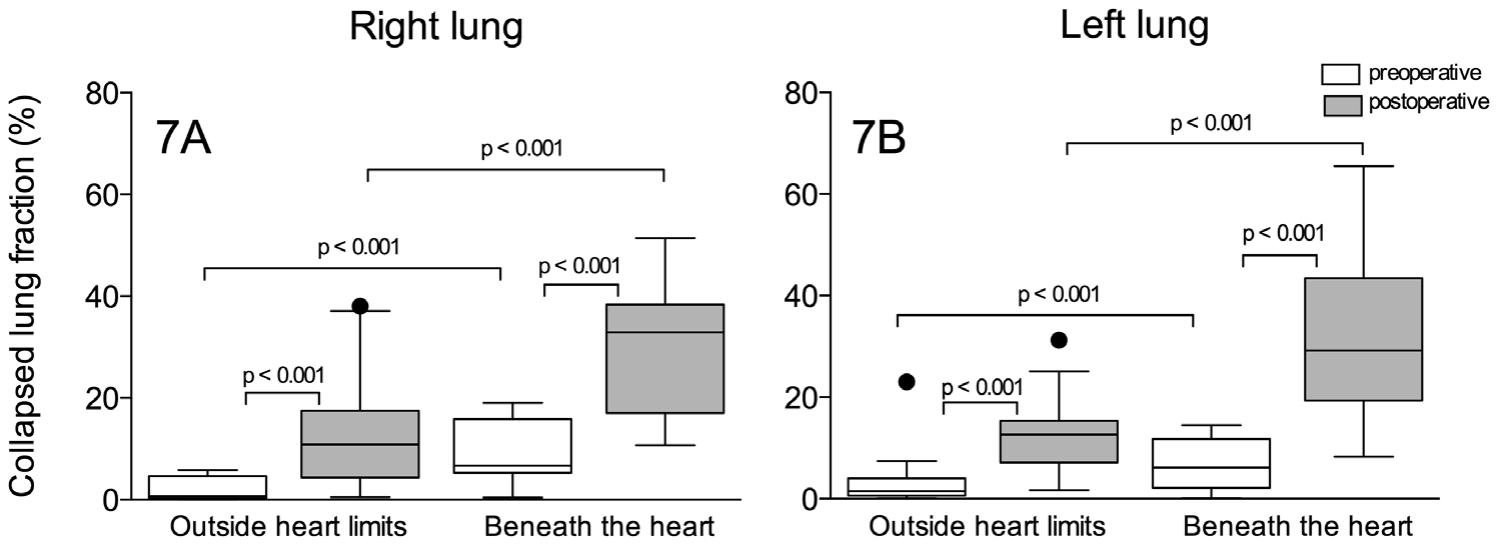
Fraction of non-aerated to overall parenchyma mass of right and left lower lobe segments located beneath the heart and outside of the heart limits before (open box-plots) and after surgery (gray box-plots). The bottom and top of the box are the lower and upper quartiles, respectively and the horizontal line within the box is the median. The whiskers represent 1.5 times the quartiles according to Tukey method.

As displayed in figure 7, the fractions of non-aerated to overall pulmonary parenchymal masses of the right and left lower lobe segments located beneath the heart were significantly greater than in the segments located outside of the heart limits, before and after surgery. In the postoperative period, the fractions of the collapsed lung parenchyma increased by 22.1% and 16.6% in the right and left respective segments of the lower lobes beneath the heart. In pulmonary segments located outside of the heart limits, the increases in the collapsed lung fraction were significantly less, reaching 6% and 4.7%, respectively.

## Discussion

This study describes several original findings: (1) CABG with CPB induced a 5-fold increase in collapsed lung parenchyma, accounting for 38% and 42% of the preoperative masses of the right and left lungs, respectively; (2) concomitantly, gas volumes of the right and left lungs decreased by 32% and 28%, respectively, whereas tissue volumes increased by 47% and 59%; (3) following surgery, the masses of the right and left heart increased by 22% and 34%, respectively, significantly increasing the orthogonal pressure exerted on subjacent lower lobes; (4) the fractions of atelectasis increased by 22% in lung segments located beneath the right cardiac chambers and by 6% in lung segments outside of the right heart limits; (5) the fractions of atelectasis increased by 17% in lung segments located beneath the left ventricle and by 5% in lung segments outside of the left heart limits; and (6) postoperative changes in lung morphology were associated with a 27% decrease in arterial oxygenation.

### Postoperative Loss of Lung Aeration

Immediate postoperative reduction of pulmonary aeration leading to hypoxemia is a highly prevalent pulmonary complication in patients undergoing CABG with CPB [4], [5], [31]–[33] and may persist for days. [3] CABG-induced atelectasis is mainly located in dependent regions close to the diaphragm. [14], [31], [32] Its extension can reach up to 35% of the overall lung parenchyma, depending on the method used for quantification. [1], [2], [8], [9] Several mechanisms have been proposed to explain the loss of pulmonary aeration following cardiac surgery: (1) the compression of caudal pulmonary segments by the weight of the abdominal content, shifting the relaxed diaphragm within the rib cage;[12] (2) absorption atelectasis resulting from the administration of a high inspired oxygen fraction;[7], [33], [34] (3) disconnection from mechanical ventilation and deflation of the lungs to facilitate surgical field exposure; and (4) surgical manipulation of intrathoracic structures. In the postoperative period, once the patient is awake and spontaneously breathing, lung collapse persists and hypoxemia worsens, although most of these mechanisms cannot be implicated. Hypoxemia reaches its nadir on the third postoperative day and remains insensitive to pain relief. [3] Therefore, other mechanisms are likely involved to explain the long-lasting postoperative hypoxemia. This study provides compelling evidence that heart compression through an increased cardiac mass plays a critical role.

Most of the previous CT studies on pulmonary alterations after CABG focused on the amount of postoperative atelectasis, ignoring postoperative alterations affecting pulmonary structures and intrathoracic organs that are anatomically related to the lungs. Recently, we reported that pulmonary tissue mass significantly increased during the postoperative period due to the accumulation of lung water in the interstitial space. The excess of lung mass that is related to CPB-induced high-permeability interstitial lung edema [35], [36] increases pleural pressure in dependent lung regions and likely plays a role in postoperative lung collapse. A similar mechanism has been described in ARDS patients: the superimposed hydrostatic pressure resulting from the increase in pulmonary tissue mass causes a progressive antero-posterior decrease in gas/tissue ratio [24], [37].

### Postoperative Increase in Cardiac Mass

If the excess lung tissue mass largely contributes to the vertical gradient of pulmonary aeration, it does not explain why the fraction of collapsed lung is greater in the lower lobe segments located beneath the heart than in the segments located outside of the cardiac limits. In fact, both areas are subjected to the same transpulmonary pressure and should be characterized by similar aeration. Physiologically, the pressure that is exerted by the heart on the lower lobes impacts regional aeration. [38], [39] Hyatt and coworkers reported that the physiological vertical gradient of transpulmonary pressure, which increases in dependent lung regions, was significantly amplified when the weight of the heart was increased by replacing blood with an equal volume of mercury in head-up dogs. [40] Such a mechanism was further confirmed in patients with cardiomegaly who were lying supine; left lower lobe ventilation was reduced by 40–50% without a concomitant reduction in regional perfusion. [21], [41] In patients with ARDS, we observed that the heart volume and mass were increased, and the heart exerted a significantly higher pressure on the lower lobes compared to normal subjects. As a result, aeration was virtually absent in segments of the lower lobes subjacent to the heart, whereas segments outside of the heart limits remained partially aerated. [15] In patients undergoing CABG, cardiac mass increased significantly in the postoperative period, and the pressures resulting from left and right cardiac protrusion increased, likely causing lung collapse of the pulmonary segments beneath the heart.

Two main mechanisms may explain the increase in cardiac mass after CABG. First, per-operative fluid loading increases both the volumes of the cardiac chambers and the cardiac index during the postoperative period. During the study period, patients received 6686 mL to compensate for fluid restriction that resulted from preoperative fasting and priming of the CPB circuit. Second, CPB and myocyte ischemia, resulting from cardioplegic arrest, induce an inflammatory reaction that increases microvascular permeability, impair myocardial lymphatic function and produce lung and myocardial interstitial edema. [42]–[44] Very likely, the increased cardiac mass observed during the postoperative period results from a combination of both mechanisms, which may vary in importance from one patient to another. Whatever mechanisms are involved, the higher pressure that is exerted by the increased cardiac mass on the lung regions beneath the heart likely explains the long-lasting loss of lung aeration during the postoperative period.

### Methodological Limitations

Some factors may introduce bias and reduce the accuracy of the CT assessment of lung aeration, heart mass and cardiac dimensions. The moment of the respiratory cycle at which the patient holds their breath determines the amount of gas present within the lungs during CT section acquisition and may impact the assessment of lung aeration. To avoid respiratory motion artifacts and changes in lung aeration resulting from CT acquisition at different lung volumes, patients were preoperatively trained to hold their breath for 15 seconds after a normal expiration. However, 3 patients breathed during postoperative CT section acquisition, rendering the analysis of the lung aeration changes impossible. Reproducible conditions of CT acquisition between pre- and postoperative periods are indirectly confirmed by the unchanged antero-posterior, sagittal and transverse dimensions of the rib cage before and after surgery.

Another source of error concerns the method that was used to calculate the pressure that is exerted by the heart on the lower lobes. Cardiac protrusion mass was divided by the subjacent linear surface that delimited the upper limit of the lung segments beneath the heart. This method of calculating cardiac superimposed pressure is rough and ignores non-orthogonal forces exerted by the heart on the pulmonary surface. The method also ignores the influence of the heart beats on lung compression. Cardiac dimensions and masses that are reported in this study were measured at different moments of the cardiac cycle and are representative of “mean” values across the cardiac cycle. As a consequence, the respiratory effects of cardiogenic oscillations and their impacts on lung aeration cannot be taken into account [45].

Lastly, the studied population comprised uncomplicated, spontaneously breathing patients who were free of ARDS or abdominal complications. It is highly likely that the presence of such complications would have amplified the loss of lung aeration resulting from cardiac compression, as previously demonstrated [15], [41].

In conclusion, following CABG, the pressure that is exerted by the heart on subjacent lung segments increases and is an important cause of the regional loss of aeration in lower lobes. This finding explains why, despite the disappearance of several factors known to induce atelectasis during anesthesia and surgery, lung collapse and hypoxemia persist for several days during the postoperative period. Further studies are necessary to assess whether maintaining the patient in a strict, half-sitting position early during the postoperative period would improve postoperative lung aeration.
